# Supplementary material for: Functional social support: A systematic review and standardized comparison of different versions of the DUFSS questionnaire using the EMPRO tool
Source: PLoS One. 2023 Sep 15;18(9):e0291635. doi: 10.1371/journal.pone.0291635 (PMC10503721; doi:10.1371/journal.pone.0291635)
Supplement: Attachment — Submitted filename: Response to Reviewers.docx [file pone.0291635.s004.docx]

Dear reviewers:

Thank you for the thorough review. We appreciate the improvements made to our manuscript.

In addition to including the contributions requested by the reviewers, considerations made by the editor regarding format and style have been reviewed and incorporated into the manuscript.

Each of the requested responses is detailed below.

**Reviewer #2:**

Dear authors,

The article is relevant, complete and valuable to both research community in social epidemiology and for accounting for social factors in any clinical study. The changes brought to the “Practical application” section seems to hit spot on to make readers understand why this study is so important. Table 5 provides a clear transparent view of the available information and the EMPRO results. I also particularly appreciated the detailed response to all our comments. This has been thoroughly done and very well argued making the review process so much easier at this stage.

--- Minor suggestions ---

**1. Competing interests - Authors might consider relying on a statement of interest rather than a statement of conflict of interest. Indeed, it is up to the reader to judge whether or not whatever is stated constitutes a conflict. For this reason, it is often recommended to state education interests and academic positions in the field, grant obtention, payed fees for consultations, participation to interest groups such as association, foundations, etc, contribution as scientific advisor for working groups, think tanks, etc., clinical activities in relation to the topic, etc. Readers slowly tend to find it suspicious if no interest are declared. Siting many interests that could constitute a better understanding of the underlying cognitive biases helps readers feel that the work is trustworthy.**

As recommended, a declaration of interests has been included.

*The main author, Cristina M Lozano-Hernández is a nurse researcher at the Foundation for Biosanitary Research and Innovation in Primary Care (FIIBAP) and at the Research Unit of the Healthcare Management of Primary Care of Madrid (GAAP), where she supports research projects in Primary Care in the local, national and European framework. She is a member of the Research Network on Chronicity, Primary Care and Prevention and Health Promotion (RICAPPS-Instituto de Salud Carlos III RD21/0016). PhD student in the Interuniversity Doctoral Program in Epidemiology and Public Health. Rey Juan Carlos University (Madrid, Spain).*

**2. Data availability: It could be seen as misleading to say all data is made available without restriction. Indeed, the working documents are not made available. It would be interesting to have the individual study assessments made using EMPRO made available. If you want to do this, consider depositing them on a repository such as on Zenodo.org**

Following your recommendation, the dataset corresponding to the evaluations of the different versions of the DUFSS questionnaire using the EMPRO tool has been published in Zenodo.

The publication is under DOI:

https://doi.org/10.5281/zenodo.8211219

**3. Line 342, the citation to the reference for Broadhead et al. still needs to be added.**

The requested reference has been included. In addition, the reference list has been revised and updated.
